# Enzyme-Free Electrochemical Nano-Immunosensor Based on Graphene Quantum Dots and Gold Nanoparticles for Cardiac Biomarker Determination

**DOI:** 10.3390/nano11030578

**Published:** 2021-02-26

**Authors:** Bhargav D. Mansuriya, Zeynep Altintas

**Affiliations:** Institute of Chemistry, Technical University of Berlin, Straße des 17. Juni 124, 10623 Berlin, Germany; b.mansuriya@campus.tu-berlin.de

**Keywords:** enzyme-free electrochemical nano-immunosensor, graphene quantum dots, gold nanoparticles, screen-printed gold electrode, biomarker detection

## Abstract

An ultrasensitive enzyme-free electrochemical nano-immunosensor based on a screen-printed gold electrode (SPGE) modified with graphene quantum dots (GQDs) and gold nanoparticles (AuNPs) was engineered to detect cardiac troponin-I (cTnI) for the early diagnosis of acute myocardial infarction (AMI). The GQDs and in-house synthesized AuNPs were implanted onto the SPGE and allowed for anti-cTnI immobilization prior to quantifying cTnI. The biomarker could be determined in a wide concentration range using square-wave voltammetry (SWV), cyclic voltammetry (CV), electron impedance spectroscopy (EIS) and amperometry. The analyses were performed in buffer, as well as in human serum, in the investigation ranges of 1–1000 and 10–1000 pg mL^−1^, respectively. The detection time ranged from 10.5–13 min, depending on the electrochemical method employed. The detection limit was calculated as 0.1 and 0.5 pg mL^−1^ for buffer and serum, respectively. The sensitivity of the immunosensor was found to be 6.81 µA cm^−2^ pg mL^−1^, whereas the binding affinity was determined to be <0.89 pM. The sensor showed high specificity for cTnI with slight responses for nonspecific biomolecules. Each step of the sensor fabrication was characterized using CV, SWV, EIS and atomic force microscopy (AFM). Moreover, AuNPs, GQDs and their nanocomposites were characterized by transmission electron microscopy (TEM) and scanning electron microscopy (SEM). This is the first immunosensor that represents the successful determination of an analyte using four different electrochemical techniques. Such a sensor could demonstrate a promising future for on-site detection of AMI with its sensitivity, cost-effectiveness, rapidity and specificity.

## 1. Introduction

Cardiovascular diseases (CVDs) are linked with blood vessels and the heart [1]. They result in nearly 33% of all deaths globally and hold significant morbidity, of which 80% of CVD deaths are due to strokes and heart attacks [2]. Those who are at a high risk of CVDs may show symptoms, such as elevated blood pressure, increased blood cholesterol and raised glucose levels, as well as induced obesity, which can be readily monitored in primary healthcare facilities. Identifying those individuals at severe risk of CVDs and assuring that they receive concise treatment can be prevented from premature deaths [3]. It is henceforth noteworthy to diagnose such patients at an initial stage that can reduce additional expenses by screening their hospital admission process and by focusing on those resources that are specifically at high-risk [1]. Therefore, prognostic biomarkers are required to be detected with minimally invasive techniques to improve the treatment of CVDs, whereas the choice of accurate, rapid, ultrasensitive and reliable sensing methods for the detection of CVDs is highly demanded to be explored [4].

In biomedicine, research for designing electrochemical immunosensors has evolved over the past decade because it is critical to selectively detect biomarkers in clinical samples for the effective diagnosis and monitoring of various diseases [5]. To meet the demand for quick, low-cost, specific and easy detection of such biomolecules in ultralow concentrations, numerous nanomaterials have garnered tremendous attention for the development of immunosensors and have been expanded to enhance the sensitivity, selectivity and reproducibility of such sensing platforms. Hence, electrochemical immunosensors hold vital features, rendering them highly appropriate for the quantification of CVD biomarkers at very low concentrations in biological fluids [6]. The exclusive merits of these sensors in terms of high sensitivity, selectivity and stability supplied by nanostructuring their sensor surface have emerged in the introduction of innovative electrochemical immunosensing strategies. Such immunosensing techniques are being developed as desirable substitutes to the conventional methods for clinically diagnosing and managing several CVDs [7].

Electrochemical sensors exhibit superior features when compared to optical and thermal sensors owing to their unique detectability, experimental simplicity and cost-effectiveness [8]. They have a prominent position among the currently accessible sensors that have reached the commercial stage and have been well-known for a wide range of important applications in the area of biomedicine [9,10]. This sensor type can function as a miniaturized device for point-of-care testing (POCT) [11,12]. To date, a number of nanomaterials-based immunosensing strategies have been reported, which possess the convincing ability for designing biosensors [13]. In this regard, nanomaterials, such as graphene quantum dots (GQDs) and gold nanoparticles (AuNPs), have prompted a particular interest in the fabrication of electrochemical immunosensors [7,13,14,15,16].

Since the past decade, various nanomaterials-based electrochemical immunosensors have been reported for the quantification of cTnI [6,13,17]. A photoelectrochemical immunosensor based on N,S−GQDs and CdS cosensitized hierarchical Zn_2_SnO_4_ cube was proposed for cTnI detection [18], wherein N,S−GQDs were implanted on the Zn_2_SnO_4_ cube, resulting in the increased photo-to-current transferability and electroconductivity. In addition, an optical sensor based on amine-functionalized GQDs was developed for cTnI determination [19]. Several other immunosensors based on GQDs were reported for sensing other cardiac biomarkers, such as cardiac myoglobin (cMyo) [20], tyrosine kinase receptor (AXL) [21] and C-reactive protein (CRP) [22]. Apart from the electrochemical immunosensors that highlighted the importance of AuNPs in their development for cTnI [11], several other biosensors working under the principle of electrochemiluminescence (ECL) [23], surface plasmon resonance (SPR) [24] and field-dependent electron transfer (FET) [25] also proved the significance of AuNPs for designing immunosensors for cTnI. All these aforementioned sensors revealed the supreme characteristics for introducing some novel immunosensing strategies based on nanomaterials for the early diagnosis of CVDs.

**Figure 1:**
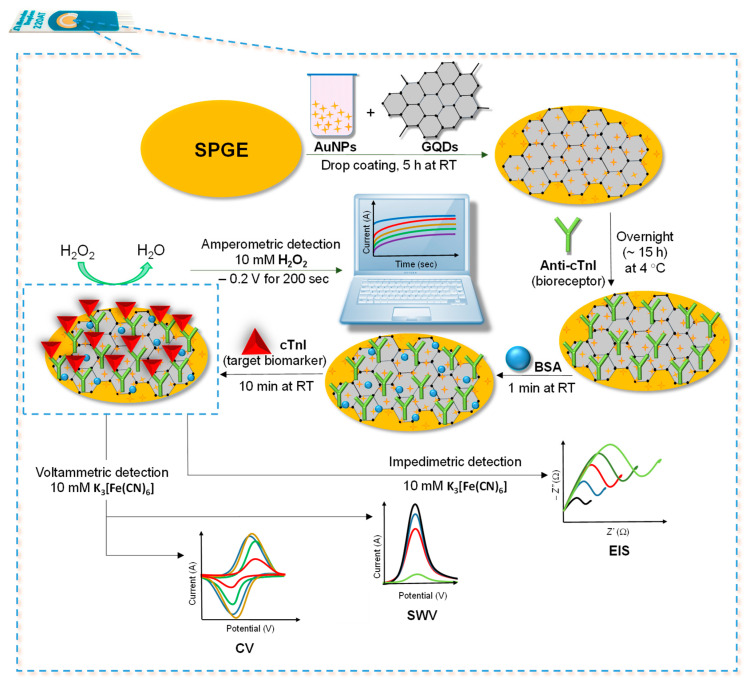
Steps involved for the fabrication of as-designed AuNPs@GQDs-modified screen-printed gold electrode (SPGE)-based label-free electrochemical immunosensor for cTnI detection.

In the current work, a GQDs and AuNPs-based screen-printed electrochemical immunosensor was constructed for the determination of cTnI as a model biomarker. The enzyme-free nano-immunosensor was fabricated by drop-coating the optimized concentrations of GQDs and AuNPs onto the screen-printed gold electrode (SPGE) surface. The layout of all the fabrication steps involved in the development of this sensor is delineated in Figure 1. AuNPs and GQDs were preferred as the potent nanomaterials owing to their marvelous features, such as good biocompatibility, signal amplifying attributes, excellent electro-catalytic activity, being responsible for the electrode surface area enhancement, abundant sites for chemical modification and cost-effectiveness [26]. The anti-cTnI antibody was then immobilized onto the AuNPs@GQDs nanocomposite layer during overnight incubation, followed by blocking of the antibody-free areas with bovine serum albumin (BSA). Individual nanomaterials, nanocomposite formation and sensor fabrication steps were characterized using several microscopic as well as electrochemical techniques. Thereafter, the anti-cTnI immobilized sensor was employed for the specific recognition of cTnI antigen, where the detection of an analyte with CV, SWV, EIS, and chronoamperometry techniques was achieved for the first time.

## 2. Materials and Methods

All chemicals and materials, electrochemical measurements, their experimental conditions (Table S1) and the related instrumentation, the cleaning protocols of the SPGE and silicon (Si) wafer, as well as the in-house synthesis of AuNPs, are provided in the Supporting Information.

To engineer the nanocomposite-modified enzyme-free electrochemical nano-immunosensor for the determination of cTnI biomarker, several parameters were investigated during the preliminary studies prior to investigating the appropriate conditions required for the establishment of bioassays. These studies involved the optimization of nanocomposite and H_2_O_2_ concentration for amperometric analysis, implanting strategies for nanocomposites onto the SPGE surface as well as anti-cTnI (cTnI- antibody) concentration. In addition, to confirm the significance of nanocomposites, amperometric responses offered by both the bare as well as nanocomposite-functionalized SPGE were measured and compared.

### 2.1. SEM and TEM Imaging of Nanomaterials

AuNPs, GQDs and their nanocomposites were analyzed using SEM and TEM. The SEM samples were prepared in absolute ethanol and drop-coated on cleaned Si wafers. The samples were then made conductive using Leitsilber (Plano GmbH, Wetzlar, Germany). SEM images were obtained using a Zeiss GeminiSEM 500 NanoVP (LEO Elektronenmikroskopie GmbH, Oberkochen, Germany) field emission scanning electron microscope at 15 kV accelerating voltage. The secondary electrons were detected with the help of an Everhart–Thornley Inlens detector. The oversampling was 16 with a line scanning rate of 20 Hz, and the magnification studies were done at 5 k, 20 k and 50 k. EDX analyses were performed using an Octane Elect detector (EDAX Ametek,Mahwah, NJ, USA) and processed with Genesis. The TEM samples were prepared in double-distilled water and visualized using bright-field and high-resolution TEM (HRTEM) with the help of a carbon-based substrate material. The microscope was set to 200 kV. The imaging software was “Digital Micrograph” from Gatan (Pleasanton, CA, USA).

### 2.2. Determination of Optimal Nanocomposite and H_2_O_2_ Concentrations

GQDs and AuNPs were employed particularly as nanozymes and signal amplifying agents in order to replace the enzymatic systems as well as to enhance the sensitivity of as-designed antibody-sensor, respectively. Considering our recent research on GQDs-based immunosensor [27], two different concentrations of GQDs (500 ppm and 1000 ppm) were examined in combination with the two distinct concentrations of AuNPs (2× dilution: 2.13 × 10^13^ particles mL^−1^ and 4× dilution: 1.06 × 10^13^ particles mL^−1^) for realizing the convenient nanocomposite concentration to be used ultimately in the sensor development based on their signal–concentration relationship. These measurements were performed using amperometry in the presence of 40 μL H_2_O_2_. All the amperometric measurements were carried out for 200 sec on the nanocomposite-coated SPGE surface. Furthermore, the concentration of H_2_O_2_ was optimized for the amperometric studies, and the influence of incubation time for H_2_O_2_ was also investigated. All experiments were performed in triplicate for the optimization of nanomaterials and H_2_O_2_ concentrations.

### 2.3. Lamination of GQDs and AuNPs on the SPGE

Three strategies were studied for filming the cleaned SPGEs with AuNPs@GQDs nanocomposite. The first approach was examined by electrodepositing the AuNPs@GQDs using multistep amperometry with 60 pulse pairs starting with 0 V for 5 s and followed by 0.9 V for 1 s. The second approach relied on the electrodeposition of AuNPs@GQDs in the presence of dopamine using multistep amperometry (60 pulse pairs, 0 V for 5 s and followed by 0.9 V for 1 s), whereas the third approach was based on the drop-coating of AuNPs@GQDs for 5 h at room temperature. Out of these three approaches, the drop-coating phenomenon was selected for the implantation of AuNPs@GQDs according to the preliminary results.

### 2.4. Optimization of Antibody Immobilization

After drop-coating the nanocomposites on the SPGE surface, the functional groups of GQDs were activated using EDC-NHS coupling chemistry. For this, a freshly prepared mixture of 0.4 M EDC and 0.1 M NHS at a 1:1 volume ratio was used for a 10 min incubation. The 40 µL of anti-cTnI antibody was then immobilized on the SPGE surface during an overnight immobilization at 4 °C. The antibody solution was prepared using 10 mM sodium acetate buffer at pH 5.5. The different concentrations of anti- cTnI (10 μg mL^−1^, 25 μg mL^−1^ and 50 μg mL^−1^) were examined to determine the optimal anti-cTnI concentration. After antibody immobilization, the SPGE surface was rinsed with PBS buffer and subsequently with double distilled water to get rid of the unbound antibody.

### 2.5. AFM Characterization of Sensor Fabrication

The stepwise fabrication of the SPGE was characterized using AFM. The AFM samples were prepared on Si wafers. These wafers were electrodeposited with 40 nm terbium and cleaned by ultrasonication (60 °C, 35 kilohertz) for 30 min in 20 mL absolute ethanol. Thereafter, they were washed with isopropyl alcohol and dried with a 1 bar nitrogen gun. AuNPs@GQDs were drop-coated on the clean wafers. The surface topography, the root mean square (RMS) roughness and cross-section values were measured by AFM NanoWizard II (JPK Instruments AG, Berlin, Germany). The measurements were performed in a dry state at room temperature in an intermittent contact mode using a TAP300 GD-G probe from Budget Sensors (Innovative Solutions Bulgaria Ltd., Sofia, Bulgaria) on cantilevers with a resonance frequency in the range of 300 ± 100 kHz. The scanning line rate was kept as 0.2 Hz. The areas of 3 μm × 3 μm were scanned and analyzed. The images were then processed by using JPKSPM Data Processing software 5.1 (JPK Instruments AG, Berlin, Germany).

### 2.6. cTnI Detection Assay in Buffer

After antibody immobilization and prior to the biomarker detection assays, the SPGEs were incubated with 100 µg mL^−1^ BSA (prepared in double distilled water) for 1 min to block the antibody-free areas on the SPGE as well as to avoid the possible nonspecific interactions during the immunoreaction. For the ultimate target determination in the buffer, a wide range of cTnI concentrations (1–1000 pg mL^−1^) was studied for the voltammetric (CV and SWV), amperometric and impedimetric (EIS) detection. Prior to the electrochemical measurements, 40 µL of cTnI was incubated with an anti-cTnI immobilized sensor surface for 10 min at room temperature. Upon immunoreaction, the SPGE surface was then washed with PBS. Afterward, the CV, SWV and EIS measurements were performed in a redox marker solution containing 10 mM K_3_[Fe(CN)_6_] and 100 mM KCl, whereas the amperometric measurements were conducted in the presence of 10 mM H_2_O_2_ solution.

### 2.7. Cross-Reactivity Studies

The specificity of the as-developed nano-immunosensor was investigated by testing the possible cross-reaction of the reference biomolecules, including NSE, D-(+)–glucose, BSA, transferrin and dopamine. All of these solutions were prepared in PBS at 1000 pg mL^−1^ concentration and were incubated for 10 min at room temperature prior to the electrochemical measurements with the SWV method in the presence of redox marker solution. For confirming the reproducibility, this study was carried out in triplicate by applying 40 µL of the sample volume in each experiment. The relative signal suppression was computed, and the results were compared.

### 2.8. cTnI Detection Assay in Human Serum Samples

To realize the potential of the as-fabricated nano-immunosensor for its clinical applicability, electrochemical detection of cTnI was investigated in human serum. For this, the biomarker was prepared in a concentration range of 10–1000 pg mL^−1^ in 50% human serum. All other parameters for the serum studies were kept similar to that for the biomarker detection in the buffer.

## 3. Results and Discussion

### 3.1. Characterization of Nanomaterials

Several characterization techniques were employed to study and understand the morphology, elemental composition, granular orientation, topography and crystallographic information of the as-selected nanomaterials. These characterization methods included UV-vis spectra in determining the size and concentration of AuNPs; SEM and TEM for examining AuNPs, GQDs as well as the nanocomposite mixture; whereas EDX spectra and XRD (X-ray diffraction) for determining their elemental composition and confirming their size with respect to their corresponding reference values, respectively.

#### 3.1.1. Characterization of AuNPs

**Figure 2:**
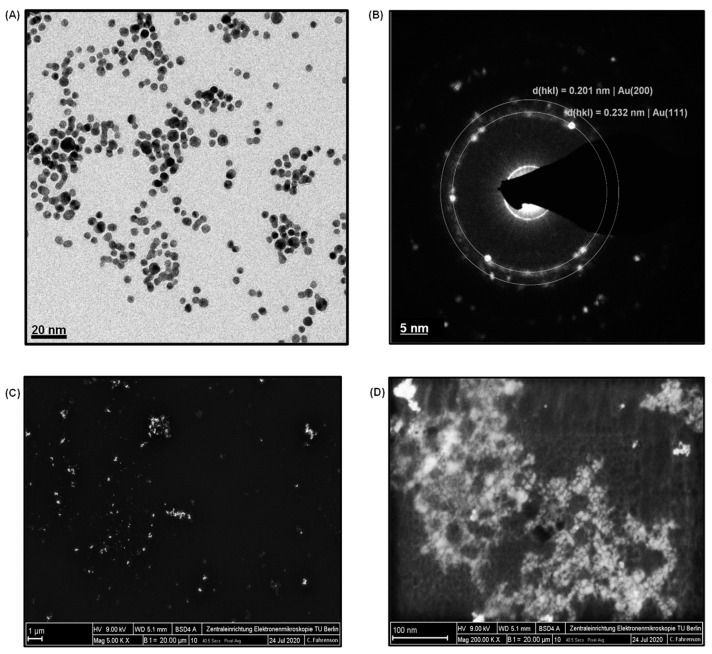
(**A**) TEM image of AuNPs. (**B**) XRD image of AuNPs. (**C**) SEM image of AuNPs at 5000× magnification. (**D**) SEM image of AuNPs at 200,000× magnification.

AuNPs were employed as the signal amplification agent to enhance the sensitivity of the cTnI nano-immunosensor. They were characterized by TEM and SEM. The size distribution of AuNPs (Figure S1A) was determined from their TEM images (Figure 2A). Spherical nanoparticles with an average diameter of 4.7 ± 2 nm were observed. Moreover, the size and the concentration of AuNPs were further calculated by UV-vis absorption studies since the absorption properties of nanoparticles depend on their size. As depicted in Figure S1B, the absorption peak (0.495) in the UV-vis spectrum was attained at 514 nm and confirmed the size range of AuNPs, which was in agreement with the literature [28]. The concentration of these in-house synthesized AuNPs was determined to be 4.27 × 10^13^ particles mL^−1^ on the basis of Beer–Lambert law [8]. Figure 2B depicts an XRD image of AuNPs, where the *d*-spacing values (i.e., inter-atomic spacing values) were recorded as 0.232 nm. This proved that the in-house synthesized AuNPs exhibited crystalline characteristics, which could fingerprint the crystalline characteristics of the standard AuNPs with 0.201 nm. The SEM analysis employed at 1 µM and 100 nm scales with magnifications of 5000× (Figure 2C) and 200,000× (Figure 2D) also confirmed the uniform size and morphology of the in-house synthesized AuNPs. Two differently diluted concentrations of AuNPs, 2× and 4× dilutions, were used in this study. These solutions included 2.13 × 10^13^ particles mL^−1^ and 1.06 × 10^13^ particles mL^−1^, respectively.

#### 3.1.2. Characterization of GQDs

**Figure 3:**
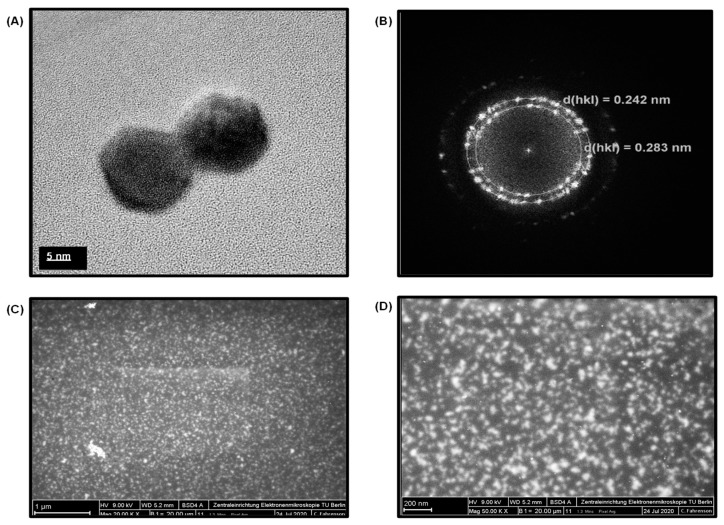
(**A**) TEM image of GQDs. (**B**) XRD image of graphene quantum dots (GQDs). (**C**) SEM image of GQDs at 20,000× magnification. (**D**) SEM image of GQDs at 50,000× magnification.

The TEM studies for GQDs divulged their size of around 8 to 10 nm (Figure 3A). Figure 3B shows the XRD image of GQDs, where the *d*-spacing values were found to be 0.283 nm. This confirmed the ideal crystalline features of the as-used GQDs with that of their standard inter-atomic *d*-spacing values (0.242 nm). In addition, SEM was performed at 1 µm and 200 nm scales with magnifications of 20,000× and 50,000×, respectively (Figure 3C,D), which confirmed the uniform size and morphology of GQDs. The EDX spectrum of GQDs exhibited their elemental composition (Figure S2). Since GQDs are carbon-based anisotropic nanomaterials, as expected, the EDX spectrum could generate a large peak of carbon and oxygen with 1748 and 238 counts at 0.28 keV and 0.53 keV, respectively. Moreover, the grid used as a background substrate for the TEM analysis of GQDs samples also contained carbon along with copper element, which produced the low as well as high-energy Cu-peaks with 260, 1442 and 220 counts at 0.95 keV, 8.02 keV, and 8.91 keV, respectively. The reason for the existence of multiple Cu-peaks is due to their large atomic radii and the availability of six different shells (i.e., Kα, Kβ, Lα, Lβ, Mα, and Mβ), which are capable of providing high-energy electrons for the EDX spectrum. Additionally, the small peaks observed at an accelerating voltage of 1.71 keV, 2.31 keV and 3.73 keV belong to silicon, sulfur and calcium, respectively. These peaks could have been appeared due to the trace amounts of such elements present within the GQD sample, resulting from the precursors used during their synthesis.

#### 3.1.3. Characterization of AuNPs@GQDs Nanocomposite

**Figure 4:**
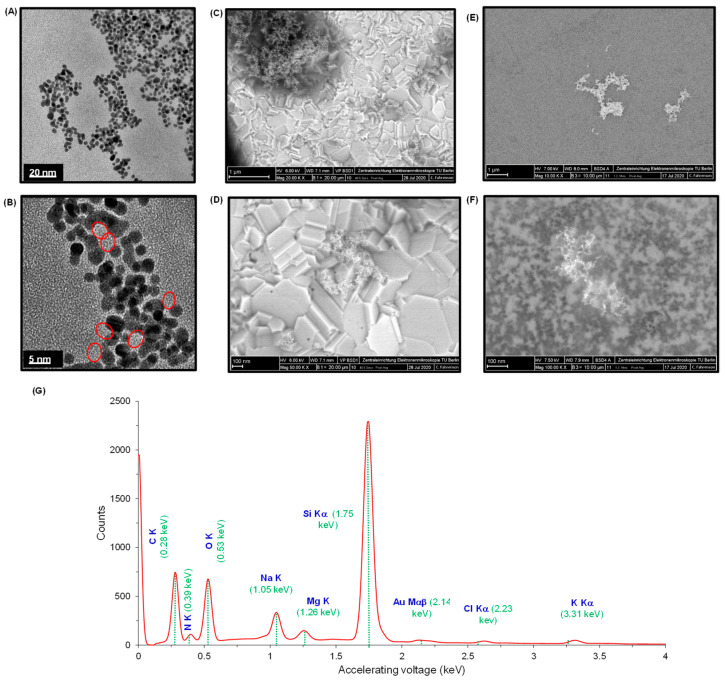
TEM images of AuNPs@GQDs at (**A**) 20 nm and (**B**) 5 nm scales. SEM images of AuNPs@GQDs/SPGE at (**C**) 1 µm and (**D**) 100 nm scales at 20,000× and 50,000× magnifications, respectively. SEM images of GQDs@AuNPs/Si-Wafer at (**E**) 1 µm and (**F**) 100 nm scales at 10,000× and 100,000× magnifications, respectively. (**G**) EDX spectrum of AuNPs@GQDs. The dotted green lines correspond to the accelerating voltage of their respective chemical elements.

The characterization of AuNPs@GQDs nanocomposite was investigated in detail in order to understand the distinct morphological features of the individual nanomaterials when combined together. Figure 4A,B represents the TEM images of nanocomposite mixture at 20 nm and 5 nm scales, which show their uniform size distribution. For SEM studies, considering the as-developed cTnI nano-immunosensor, the nanocomposite mixture was initially characterized onto the SPGE surface (Figure 4C,D) at 1 µm and 100 nm scales at 20,000× and 50,000× magnifications, respectively. Although the GQDs appeared in the form of cluster, AuNPs were still not clearly visible as expected. Herein, the involvement of SPGE containing gold as a working electrode could obstruct the differentiation of AuNPs from GQDs. To overcome this problem, a silicon wafer was used as a background substrate for a better observation. The presence of both the AuNPs and GQDs together in a nanocomposite mixture was clearly confirmed from the SEM images (Figure 4E,F) captured at 1 µm and 100 nm scales at 10,000× and 100,000× magnifications, respectively. Here, AuNPs were appeared as dark granular particles with a uniform size distribution, whereas the cluster of GQDs appeared in the form of an island.

To determine the chemical composition residing in the nanocomposite mixture, the EDX spectrum (Figure 4G) was interpreted for the AuNPs@GQDs/Si wafer. The carbon and oxygen peaks, respectively at 0.28 keV and 0.53 keV, confirmed the presence of GQDs, whereas the existence of AuNPs was realized from the Au-peak that appeared at 2.14 keV. Since the mixture containing AuNPs and GQDs was drop-coated on Si wafer, the EDX spectrum revealed a sharp Si-peak at 1.75 keV. In addition, the emergence of other peaks at 0.39 keV, 1.05 keV, 1.26 keV, 2.23 keV and 3.31 keV indicated the presence of nitrogen, sodium, magnesium, chlorine and potassium, respectively. The reason behind the appearance of these peaks could be attributed to the presence of inorganic salts in the nanocomposite mixture, resulting from the precursors used during their respective chemical syntheses.

### 3.2. Determination of Optimal Nanocomposite and H_2_O_2_ Concentrations

**Figure 5:**
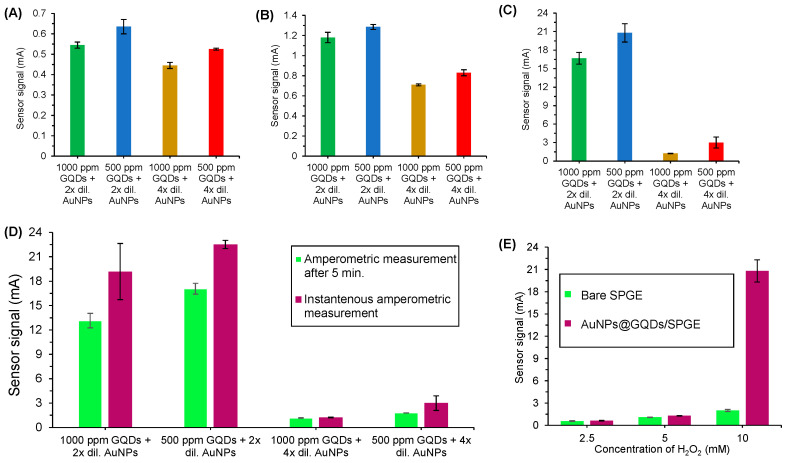
Signal–concentration relationship for various concentrations of nanocomposite mixture using (**A**) 2.5 mM H_2_O_2_ (**B**) 5.0 mM H_2_O_2_ (**C**) 10 mM H_2_O_2_, obtained by amperometric measurements. (**D**) Signal-nanocomposite relationship in amperometric measurements with and without waiting for 5 min after the application of 10 mM H_2_O_2_. (**E**) Comparison of the signal–concentration relationship between bare SPGE and AuNPs@GQDs/SPGE (*n* = 3). 2× and 4× dilutions of AuNP solutions include 2.13 × 10^13^ particles mL^−1^ and 1.06 × 10^13^ particles mL^−1^, respectively.

Herein, GQDs and AuNPs were employed particularly as nanozymes and signal amplifying agents in order to replace the enzymatic systems as well as to improve the sensitivity of the developed nano-immunosensor, respectively. Two different concentrations of GQDs (500 ppm and 1000 ppm) were examined in combination with two different concentrations of AuNPs (2× and 4× dilutions) for testing the convenient nanocomposite to be used ultimately in the sensor development based on their signal–concentration relationship. The measurements were performed using amperometry in the presence of 40 µL H_2_O_2_ for 200 sec on the nanocomposite coated SPGE surface. As depicted in Figure 5A–C, the amperometric measurements were carried out using three different concentrations of H_2_O_2_ (2.5 mM, 5.0 mM and 10 mM) for all the possible combinations of the aforementioned nanocomposite concentrations. From these analyses, 10 mM H_2_O_2_ could generate extremely elevated sensor signals (up to 20.8 mA) for the nanocomposite mixtures, whereas 2.5 mM and 5 mM H_2_O_2_ could generate signal response up to 0.63 mA and 1.24 mA, respectively. The optimal nanocomposite mixture, containing 500 ppm GQDs and 2.13 × 10^13^ particles mL^−1^, could generate 33 and 17 times higher signals in comparison to those with 2.5 mM and 5 mM H_2_O_2_, respectively. Of note, 1000 ppm and 500 ppm GQDs did not exhibit a significant difference in the signal response, despite being slightly higher in the latter case. Hence, 500 ppm GQDs and 2.13 × 10^13^ particles mL^−1^ AuNPs (2× dilution) were selected for further experimentations and bioassays.

To examine the importance of time for amperometric measurements after the addition of 10 mM H_2_O_2_ on the SPGE surface, further experiments were carried out using the already optimized nanocomposite concentration. The amperometric measurements were performed after 5 min incubation of 10 mM H_2_O_2_ on the surface (Figure 5D). It was observed that the incubation is not necessary since the resultant signal response was reduced to almost 17% upon waiting for 5 min prior to the measurements. Henceforth, the amperometric bioassays were conducted immediately after adding H_2_O_2_. Considering the synergistic role offered by GQDs as well as AuNPs and to further realize their significance, an amperometric analysis was also executed on a bare SPGE surface. As expected, AuNPs@GQDs/SPGE surface could produce a much higher signal, which was almost 8-fold higher than the bare SPGE surface (Figure 5E), implying that the intimate electronic interactions between nanocomposites and SPGE provided the amplified sensor signals and increased the electro-catalytic activity that led them into the exceeding active electro-catalysts for the reduction of H_2_O_2_.

GQDs are nanomaterials with a unique sp^2^ and sp^3^ hybrid structure. Being a zero-dimensional (0D) carbon-based nanomaterial, the chemical structure of GQD constitutes 42 sp^2^ and 7 sp^3^ carbon atoms that makes the ratio of carbon in sp^2^ and sp^3^ hybridization = 6:1. In the comparison between carbon-based nanomaterials, i.e., graphene and carbon nanotube, GQDs have a higher number of carboxyl groups, high conductivity, and high surface-to-volume ratio. The carboxylic moieties at GQDs edges enhance water dispersibility and the formation of a complex with various compounds. One of the problems that limit the electrical properties of GQDs is the lack of a bandgap in graphene. To address this problem, we formed a hybrid nanocomposite combining metal-based conductor nanoparticles (AuNPs) with GQDs to obtain superior electrical conductivity and excellent catalytic activity in the construction of an electrochemical sensor, where the composite catalyzes the reaction of H_2_O_2_ to H_2_. Further studies to elucidate the dominant mechanisms of these catalytic reactions are still undergoing.

### 3.3. Selection of Approach to Implant AuNPs@GQDs on the SPGE

Three strategies introduced in Section 2.3 were investigated for the nanocomposite implantation onto the SPGE. Out of these, the sensor signal after drop-coating for 5 h was found to be significantly higher (~10-fold) than those after electrodepositing nanocomposites, even in the existence of dopamine (Figure S3). Electrodeposition of dopamine along with certain nanomaterials usually leads to the formation of a film on metallic bioelectrodes [29]. Interestingly, electrodepositing AuNPs and GQDs on the SPGE surface (both in the absence and presence of dopamine) could not generate considerable sensor signals. This means that such an electrochemical modification of as-selected nanocomposites is inappropriate for bio-electrode application since this approach could not enable fine control over deposition sites and the extent of deposition of AuNPs and GQDs on the SPGE surface. In addition, the presence of the electrically insulating polydopamine layer could degrade the electrical properties of AuNPs and GQDs when employed for bio-electrode modification. Therefore, the drop-coating approach was selected for fabricating the sensor as well as for performing the biodetection assays.

### 3.4. Antibody Immobilization

Prior to developing the bioassays, three different concentrations of cTnI antibody (10 µg mL^−1^, 25 µg mL^−1^ and 50 µg mL^−1^) were investigated. As depicted in Figure S4, the square-wave voltammetric signals for 10 µg mL^−1^, 25 µg mL^−1^ and 50 µg mL^−1^ of anti-cTnI were suppressed up to 25.99%, 54.05% and 54.55%, respectively, by accounting their respective nanocomposite-modified SPGE signal response as the reference peaks. It was clearly noticeable that 10 µg mL^−1^ of anti-cTnI could not be sufficient for the anti-cTnI immobilization when compared to the other concentrations. Additionally, considering the negligible difference between the voltammetric signal responses obtained for 25 µg mL^−1^ and 50 µg mL^−1^ of anti-cTnI, 25 µg mL^−1^ of anti-cTnI was chosen as the optimal antibody concentration for conducting the bioassays to not increase the ultimate cost of the as-designed nanocomposite-based electrochemical immunosensor.

### 3.5. Sensor Fabrication and Characterization

**Figure 6:**
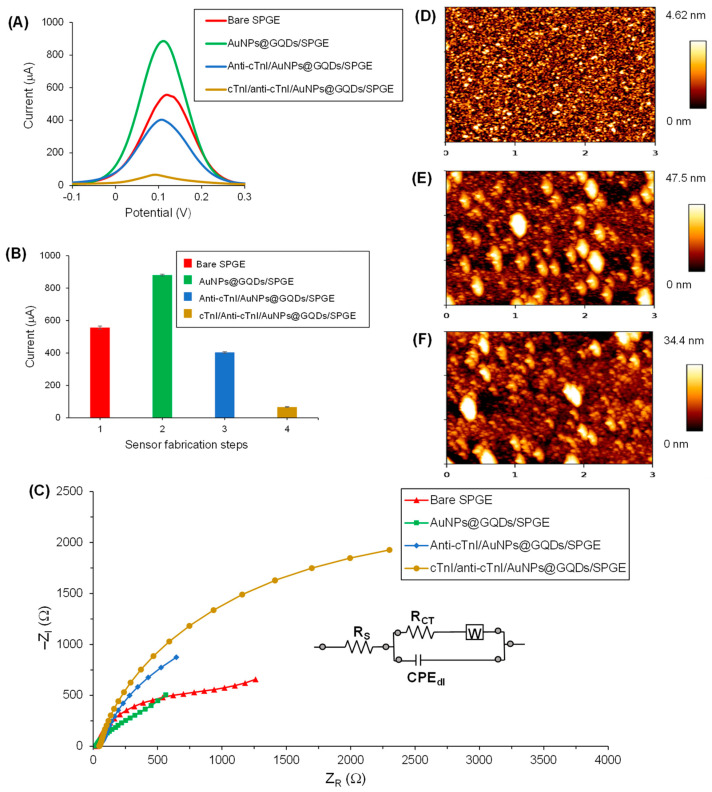
(**A**) Square-wave voltammograms recorded using redox marker containing 10 mM K_3_[Fe(CN)_6_] and 0.1 M KCl for the bare SPGE (red curve), AuNPs@GQDs/SPGE (green curve) anti-cTnI/AuNPs@ GQDs/SPGE (blue curve) and cTnI/anti-cTnI/AuNPs@GQDs/SPGE (brown curve). (**B**) Comparison of the signal responses generated by each fabrication step. Step 4 involved 100 pg mL^−1^ cTnI binding on the nanocomposite sensor. (*n* = 3). (**C**) Nyquist plots recorded using redox marker containing 10 mM K_3_[Fe(CN)_6_] and 0.1 M KCl for the bare SPGE (red curve), AuNPs@GQDs/SPGE (green curve), anti-cTnI/AuNPs@ GQDs/SPGE (blue curve) and cTnI/anti-cTnI/AuNPs@GQDs/SPGE (brown curve) using EIS. Concentration of cTnI: 100 pg mL^−1^. The inset shows the circuit model employed for the curve fittings. AFM 2D height images for (**D**) bare Si wafer, (**E**) AuNPs@GQDs/Si wafer and (**F**) anti-cTnI/AuNPs@ GQDs/Si wafer.

The assembly of the AuNPs@GQDs-modified SPGE, immobilization of the anti-cTnI antibody and its hybridization with the target cTnI to form cTnI/anti-cTnI/AuNPs@GQDs/SPGE, as well as its electrochemical detection, is portrayed in Figure 1. The SPGE containing gold as a counter electrode and working electrode with a surface area of 0.11 cm^2^ as well as silver as reference electrode was modified for the fabrication of AuNPs@GQDs-based electrochemical immunosensor for cTnI quantification. SWV and EIS techniques were employed for the characterization of each sensor fabrication step, including plasma cleaning of the SPGE, drop-coating of AuNPs@GQDs on the SPGE, immobilization of anti-cTnI on AuNPs@GQDs/SPGE, and binding of cTnI onto anti-cTnI/AuNPs@GQDs/SPGE surface after 10 min incubation time. As shown in Figure 6A,B, the peak current of SWV curves significantly increased after drop-coating the AuNPs@GQDs, suggesting the successful implantation of nanocomposites onto the SPGE surface. After depositing the SPGE surface with nanocomposites, the resultant signal amplification was due to the increase in a surface area, which would provide a large free-room for anti-cTnI immobilization. Thereafter, upon incubating the anti-cTnI antibody on AuNPs@GQDs/SPGE surface, the sensor signal remarkably decreased, confirming the successful immobilization of anti-cTnI onto AuNPs@GQDs/SPGE surface. Subsequently, upon binding of 100 pg mL^−1^ cTnI onto anti-cTnI/AuNPs@GQDs/SPGE surface, the SWV peak further reduced, indicating the presence of cTnI antigen via immunoreaction. Furthermore, a similar trend was observed in the case of CV as well (Figure S5). Although voltammetric studies were performed in triplicate for the validation of reproducibility, EIS was also conducted as a second method to double-check the successful fabrication of as-constructed AuNPs@GQDs-based electrochemical nano-immunosensor. It was observed that the results generated by the SWV method were in good agreement with those obtained by EIS, where the corresponding Nyquist plots for each step were analyzed, as shown in Figure 6C. Herein, the imaginary part of impedance, i.e., “−Z_I_ (Ω)” was plotted against the real part of impedance, i.e., “Z_R_ (Ω)” to obtain Nyquist plots. The curve fitting was accomplished using the Randles equivalent circuit (Figure 6C, inset), which consists of several parameters, such as constant solution resistance (Rs), double layer capacitance (Cdl), electron transfer resistance (R_CT_) that equals to the semicircular diameter of EIS as well as the Warburg impedance (W) [8]. Since R_CT_ governs the electron transfer kinetics of the redox probe at the electrode interface, it was observed that, when nanocomposites were drop-coated onto the SPGE surface, the R_CT_ values were significantly lowered, suggesting the efficient implantation of nanocomposites [6]. Subsequently, upon adsorbing an anti-cTnI antibody on AuNPs@GQDs/SPGE surface, it retarded the electron transferability between the electrochemical double layer and the redox probe, which led to the elevated R_CT_ values for the redox probe to access the electrochemical double-layer. These R_CT_ values were further increased to a significant extent after the incubation of cTnI on the anti-cTnI/AuNPs@GQDs/SPGE surface, which again confirmed the progressive binding of the target biomarker.

The sensor fabrication steps were also characterized by AFM (Figure 6D–F and Figure S6). The surface topographies, root-mean-square (RMS) roughness and 3D height images were captured for bare Si wafer, AuNPs@GQDs/Si wafer and anti-cTnI/AuNPs@GQDs/Si wafer at 3 μm × 3 μm areas. The bare surface of the clean Si wafer showed a highly uniform and smooth surface with 2D height and RMS roughness value of 4.62 nm (Figure 6D) and 1.03 ± 0.09 nm (Figure S6A), respectively. After drop-coating AuNPs and GQDs onto Si wafer for 5 h, these values were elevated significantly up to 47.5 nm (Figure 6E) and 11.38 ± 1.63 nm (Figure S6B), suggesting that the nanocomposites were successfully implanted on the surface of the Si wafer. At this stage, clusters of nanocomposites with height values of 20−40 nm were observed. Similarly, these clusters were also noticed in SEM images in the form of islands (Figure 4E,F). Subsequently, upon immobilizing anti-cTnI onto AuNPs@GQDs/Si wafer, the 2D height and RMS roughness values were reduced to 34.4 nm (Figure 6F) and 8.16 ± 0.78 nm (Figure S6C), indicating the alteration in the surface morphology. This phenomenon can be explained by anti-cTnI immobilization on the edges and corners of the surface created by the clusters of the nanocomposite, leading to a decreased RMS roughness values, which can also be observed in the corresponding cross-sectional height profiles (Figure S6).

### 3.6. cTnI Bioassay in Buffer

The determination of cTnI was achieved by SWV, CV, EIS, and amperometry in the concentration range of 1–1000 pg mL^−1^. This investigation range was selected by taking 0.04 ng mL^−1^ into consideration since it is the universal threshold value for cTnI in humans [30]. Beyond this threshold level, an individual may develop a high-risk for AMI and similar other CVDs.

**Figure 7:**
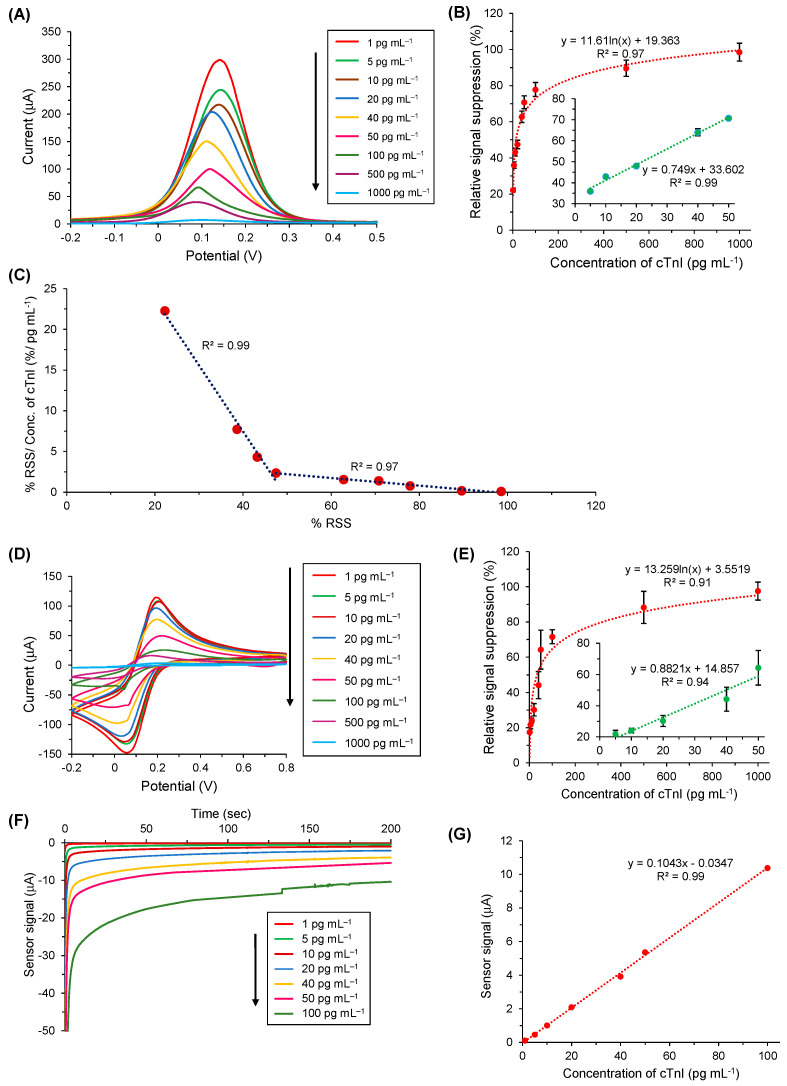
(**A**) Square-wave voltammograms of target binding with nine different cTnI concentrations (1–1000 pg mL^−1^) in PBS. (**B**) Overall results of concentration-dependent cTnI bio-assay using SWV (*n* = 3). The inset: linear regression for five concentrations of cTnI (5–50 pg mL^−1^). (**C**) Scatchard plot showing a bilinear regime with K_d_ values of 0.05 pM for concentrations below 40 pg mL^−1^ of cTnI and 0.89 pM for all other concentrations exceeding 40 pg mL^−1^ of cTnI. **(D**) Cyclic voltammograms of target binding with nine different cTnI concentrations (1–1000 pg mL^−1^) in PBS. (**E**) Overall results of concentration-dependent cTnI bio-assay using CV (*n* = 3). The inset: linear regression for five concentrations of cTnI (5–50 pg mL^−1^). (**F**) Real-time amperometric measurements were recorded for seven different concentrations of cTnI (1–100 pg mL^−1^) using 10 mM H_2_O_2_. (**G**) Overall amperometric results for cTnI binding in the concentration range of 1–100 pg mL^−1^ in PBS (*n* = 3).

The detection assay for cTnI conducted via the SWV method is displayed in Figure 7A, which clearly demonstrated that, with the increase in cTnI concentrations on anti-cTnI/ AuNPs@GQDs/SPGE surface, the SWV peaks decreased. Figure 7B depicts the relative signal reduction of cTnI concentrations from 1 to 1000 pg mL^−1^ with respect to the SWV signal response generated by anti-cTnI/AuNPs@GQDs/SPGE, confirming that the relative signal suppression was directly proportional to the cTnI concentrations. Moreover, it was found that there existed linearity from 5 to 50 pg mL^−1^ of cTnI, revealing the R^2^ value of 0.99 (Figure 7B, inset), whereas, beyond 50 pg mL^−1^, it resulted in a saturation. In addition to performing the SWV studies in triplicate to assure the reproducibility for the voltammetric detection of cTnI, CV was also employed for the same concentration range to cross-verify the results (Figure 7D,E). The results obtained by CV for the target analyte quantification were found to be in good agreement with those attained by SWV.

To determine the binding kinetics between antibody and the target analyte, dissociation constant (K_d_) was calculated by the slopes (m) obtained from the Scatchard plot (Figure 7C) [31]. On the basis of Equation (1), the two different K_d_ values were found as 0.05 pM and 0.89 pM for two concentration ranges of cTnI, i.e., 1–40 pg mL^−1^ and 40–1000 pg mL^−1^, respectively:

(1) $$Kd=-\frac{1}{m}$$

Using Equations (2) and (3), the sensitivity (*S*) and the limit of detection (LOD) were calculated, respectively as 6.81 μA cm^−2^ pg mL^−1^ and 0.1 pg mL^−1^, where m is the slope, A is the lateral surface area of working electrode (0.11 cm^2^), and *s* is the standard deviation [13].

(2) $$S=\frac{m}{A}$$

(3) $$\mathrm{LOD}=3\times \frac{s}{S}$$

For the label-free amperometric detection of cTnI, the quantification of cTnI was based on the reduction of H_2_O_2_ by GQDs, where the amount of cTnI was directly proportional to the degree of electron transfer of the GQDs grafted on the SPGE. Therefore, as shown in Figure 7F, the signal response of the as-designed immunosensor was in correlation with the cTnI concentration, ranging from 1 to 100 pg mL^−1^. Figure 7G represents the corresponding overall signal–concentration relationship generated by amperometric bio-assay in the buffer, which confirmed the linear regression with an R^2^ value of 0.99 for the investigation range.

The impedimetric analysis (Figure S7) also revealed the successful pattern for the cTnI detection similar to that of the voltammetric and amperometric analysis. The results obtained from EIS were studied by plotting the Nyquist plots and circuit model for curve fitting as explained under Section 3.5. Herein, when cTnI molecules were introduced from 1 to 1000 pg mL^−1^ onto the anti-cTnI/AuNPs@GQDs/SPGE surface, their respective R_CT_ values were elevated due to the hampered electron transferability, generating the larger semicircles in the Nyquist plots with the gradual increase in the cTnI concentration.

### 3.7. Cross-Reactivity Studies

**Figure 8:**
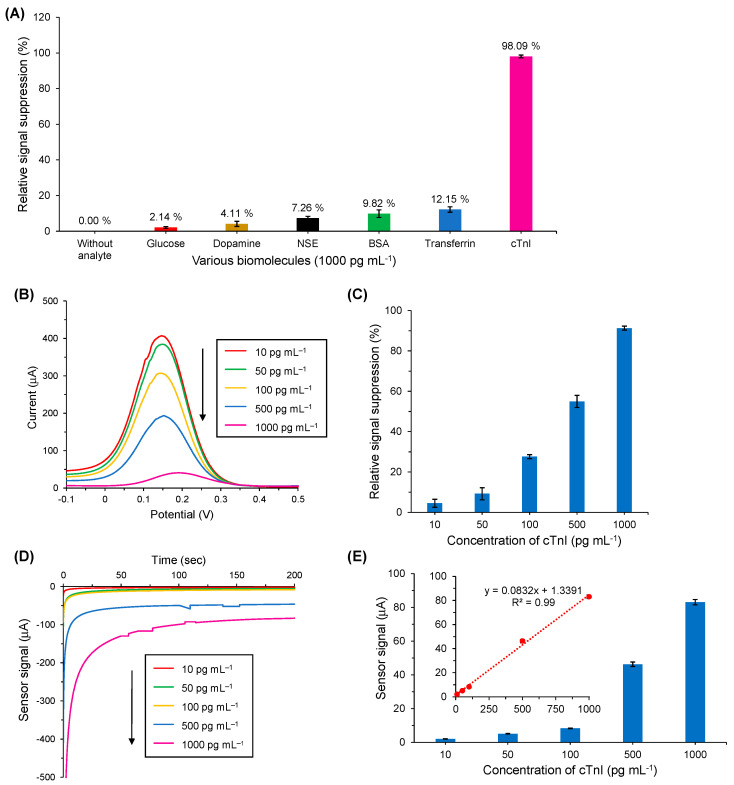
(**A**) Cross-reactivity tests for cTnI specific antibody sensor (*n* = 3). All analytes were tested at equal concentration. (**B**) Square-wave voltammograms recorded using a redox marker containing 10 mM K_3_[Fe(CN)_6_] and 0.1 M KCl for the target binding with anti-cTnI/AuNPs@GQDs/SPGE by preparing five different concentrations of cTnI (10–1000 pg mL^−1^) in human serum. (**C**) Overall results of SWV-based cTnI biodetection in human serum using the antibody sensor (*n* = 3). (**D**). Real-time amperometric measurements recorded using 10 mM H_2_O_2_ for the target binding with anti-cTnI/AuNPs@GQDs/SPGE by preparing five different concentrations of cTnI (10–1000 pg mL^−1^) in human serum. (**E**) Overall results of amperometry-based cTnI biodetection in human serum. The inset shows linear regression of signal–concentration relationship for 10–1000 pg mL^−1^ cTnI with an R^2^ value of 0.99.

To determine the specificity of the as-developed nanocomposite-based electrochemical antibody sensor for cTnI detection, the interaction of various nonspecific biomolecules with the cTnI-specific anti-cTnI immobilized AuNPs@GQDs/SPGE surfaces was examined. Herein, cTnI was tested as the target analyte, whereas human transferrin, NSE, dopamine, BSA and glucose were investigated as the nonspecific molecules (Figure 8A). All these analyte samples were incubated separately on anti-cTnI/AuNPs@GQDs/SPGE for 10 min at room temperature with a fixed concentration of 1000 pg mL^−1^, and the experiments were repeated in triplicate to ensure the reproducibility and reliability of the data.

The nonspecific binding responses of glucose, dopamine, NSE, BSA and transferrin were calculated as 2.14%, 4.11%, 7.26%, 9.86% and 12.15%, respectively. On the other hand, cTnI binding at the same concentration resulted in 98.09% signal suppression. This clearly implies that the as-designed immunosensor is highly specific for the target analyte and has shown a significant affinity towards the cTnI biomarker.

### 3.8. cTnI Bioassay in Human Serum

In order to realize the clinical applicability of the as-engineered AuNPs@GQDs electrochemical immunosensor, its performance in human serum was also studied, where the cTnI was determined in the linear concentration range from 10 to 1000 pg mL^−1^ using SWV, CV and amperometry methods. As shown in Figure 8B,C, the SWV peaks were decreased with the increase in cTnI concentrations in a linear fashion with an R^2^ value of 0.97. The CV results (Figure S8) showed a similar pattern for cTnI detection when compared to the SWV results. However, both of these voltammetric detection techniques for human serum studies revealed lower % RSS values than those for the buffer studies. The results achieved with amperometry (Figure 8D,E) were also found to be in accordance with the voltammetric studies, revealing an R^2^ value of 0.99 for the signal–concentration relationship. The LOD for human serum studies was calculated as 0.5 pg mL^−1^ on the basis of Equations (2) and (3). These results have shown a promising future for the developed nano-immunosensor to be used in the real world to diagnose patients with AMI.

The superior features of the current work are highlighted in Table S2 by comparing the other nanomaterial-functionalized electrochemical immunosensors for cTnI detection. For instance, Ahammad et al. achieved the detection of cTnI with AuNPs-modified ITO electrode, where they could measure the biomarker with the CV method in human serum with an LOD of 1000 pg mL^−1^ [13]. Better LODs (100 pg mL^−1^, 7 pg mL^−1^) were reported in human plasma with PdNPs- and PtNPs-modified SPEs, respectively, using SWV and CV methods [4]. Singh et al. were able to detect the biomarker using CV and EIS methods with an LOD of 5 pg mL^−1^, where patterned mesoporous nickel vanadate hollow-nanosphere-modified chitosan (Ch-Ni_3_V_2_O_8_) was integrated into a microfluidic electrochemical biosensor [6]. Another study reported the GQDs/PAMAM-modified immunosensor for cTnI detection, where the detection was achieved by CV and DPV methods in a wide concentration range of 10^−6^–10 ng mL^−1^ [16]. In the current work, we have shown the highly sensitive detection of cTnI with four major electrochemical techniques (SWV, CV, EIS and amperometry) for the first time by following the most economical and less complicated sensor fabrication approach. Of note, the developed sensor has exhibited a better specificity than most of the other works. The detection times for cTnI were 10.5 min, 11 min, 12 min and 13 min through SWV, CV, EIS and amperometric methods, respectively. Additionally, cTnI could be quantified over the wide linear ranges in a buffer (1–1000 pg mL^−1^) as well in human serum samples (10–1000 pg mL^−1^) with LODs of 0.1 pg mL^−1^ and 0.5 pg mL^−1^, respectively.

## 4. Conclusions

In this study, a novel electrochemical nano-immunosensor based on AuNPs and GQDs was developed using an economical, rapid and user-friendly approach. The as-fabricated sensor allows detecting the cTnI biomarker with high sensitivity using SWV, CV, EIS, and amperometry techniques. This enzyme-free electrochemical quantification of cTnI was effectively achieved on an anti-cTnI/AuNPs@GQDs/SPGE system by investigating a wide concentration of 1–1000 pg mL^−1^ cTnI in a buffer and 10–1000 pg mL^−1^ in human serum samples. The detection times were recorded between 10.5 to 13 min, depending on the electrochemical method used. Fabrication steps of the immunosensor development were characterized by CV, SWV, EIS and AFM studies, whereas the morphology, size distribution, elemental composition, granular orientation, topography and crystallographic information of AuNPs, GQDs and their nanocomposite were characterized by TEM, SEM, XRD as well as EDX. The dissociation constant was found to be < 0.89 pM, highlighting the high-affinity of the sensor towards cTnI. The lowest detection limit was computed as 0.1 pg mL^−1^ and 0.5 pg mL^−1^ for buffer and serum samples, respectively. The sensitivity of this immunosensor was found to be 6.81 µA cm^−2^ pg mL^−1^. The cross-reactivity test with five reference biomolecules confirmed the high specificity of the immunosensor for cTnI. Considering the promising results achieved, this cost-effective AuNPs@GQDs-based immunosensor can be an attractive analytical tool that may pave the way for the detection of cTnI when applied in clinical laboratories to quantify cTnI in human serum samples for the early diagnosis and monitoring of AMI.

## Data Availability

The data presented in this study are available on request from the corresponding author.

## Supplementary Materials

The following are available online at https://www.mdpi.com/2079-4991/11/3/578/s1, Table S1: Parameters for voltammetric, impedimetric and amperometric techniques, Table S2: Comparison of the current work with some relevant studies in the field, Figure S1: Size distribution histogram of AuNPs and UV-vis absorption spectrum of AuNPs, Figure S2: EDX spectrum of GQDs, Figure S3: Comparison of the signal response for different implanting approaches, Figure S4: Optimization of antibody concentration and immobilization, Figure S5: Characterization of sensor fabrication using CV technique, Figure S6: AFM characterization of sensor fabrication steps, Figure S7: Detection of cTnI in PBS buffer using EIS technique, Figure S8: Detection of cTnI in human serum using CV technique.
