# Supplementary material for: Enzyme-Free Electrochemical Nano-Immunosensor Based on Graphene Quantum Dots and Gold Nanoparticles for Cardiac Biomarker Determination
Source: Nanomaterials (Basel). 2021 Feb 26;11(3):578. doi: 10.3390/nano11030578 (PMC7996554; doi:10.3390/nano11030578)
Supplement: Supplementary file 1 [file nanomaterials-11-00578-s001.pdf]

Nanomaterials

SUPPORTING INFORMATION

Enzyme-Free Electrochemical Nano-Immunosensor Based on Graphene Quantum Dots and Gold Nanoparticles for Cardiac Biomarker Determination

Bhargav D. Mansuriya, Zeynep Altintas*

Technical University of Berlin, Straße des 17. Juni 124, Berlin 10623, Germany

***Corresponding author: Z. Altintas

Tel: +49 30 314 23727

Fax: +49 30 314 79552

E-mail: zeynep.altintas@tu-berlin.de

Table of Contents

Materials, chemicals and reagents……………………………………………………..……………3

Synthesis of gold nanoparticles …...4

SPGEs and their cleaning procedure…..4

Instrumentation and electrochemical analysis……………………….……………………..…….5

Table S1. Parameters for voltammetric, impedimetric and amperometric techniques……......…..6

Table S2. Comparison of the current work with some relevant studies in the field………………6

Fig. S1. Size distribution histogram of AuNPs and UV-Vis absorption spectrum of AuNPs…....7

Fig. S2. EDX spectrum of GQDs. …………..……………………………………...…………...7

Fig. S3. Comparison of the signal response for different implanting approaches ………......……8

Fig. S4. Optimization of antibody concentration and immobilization…..……..………..………..9

Fig. S5. Characterization of sensor fabrication using CV technique…….…………………………..10

Fig. S6. AFM characterization of sensor fabrication steps ………….…………………………...11

Fig. S7. Detection of cTnI in PBS buffer using EIS technique……………..…...…………………12

Fig. S8. Detection of cTnI in human serum using CV technique………………………………...12

References...13

Experimental section

Materials, chemicals and reagents

GQDs (blue luminescent) were purchased from Sigma Aldrich, Germany. Gold (III) chloride trihydrate (Tetrachloroauric acid, $HAuCl_4$, M.W.: 393.83 g mol^{-1}, Sigma Aldrich); Potassium carbonate: (K_2CO_3, M.W.: 138.21 g mol^{-1}, Sigma Aldrich); Tannic acid ($C_{76}H_{52}O_{46}$, M.W.: 1701.19 g mol^{-1}, Sigma Aldrich) and sodium citrate tribasic dehydrate ($C_6H_5Na_3O_7.2H_2O$, M.W.: 294.1 g mol^{-1}, Sigma Aldrich) were used to synthesize AuNPs. The functional groups (i.e., carboxyl group) of GQDs were activated using 1-ethyl-3-(-3-dimethylaminopropyl) carbodiimide hydrochloride (EDC-HCl, $C_8H_{17}N_3$.HCl, M.W.: 191.70 g mol^{-1}, Novabiochem) and N-hydroxysuccinimide (NHS, 98%, $C_4H_5NO_3$, M.W.: 115.09 g mol^{-1}, Sigma Aldrich). Phosphate buffer saline (PBS) tablets (Sigma Aldrich) and sodium acetate-1-^{13}C (≥99%, ^{13}C CH_3NaO_2, MW: 83.03 g mol^{-1}, Sigma Aldrich) were used to prepare the buffer solutions. Hydrochloric acid (36.5-38%, HCl, MW: 36.46 g mol^{-1}, Sigma Aldrich) and sodium hydroxide (99%, NaOH, MW: 40 g mol^{-1}, VWR) were used to adjust pH of sodium acetate buffer solution. Double–distilled ultrapure water was obtained from a Millipore Direct–Q® 3 UV (Millipore, Germany). Voltammetric (CV and SWV) and impedimetric (EIS) measurements were conducted for the characterization of immunosensor as well as for the target determination, with a redox marker made of potassium hexaferricyanide (≥99%, $K_3[Fe(CN)_6]$, MW: 329.26 g mol^{-1}, Carl Roth) and potassium chloride (≥99.9%, KCl, MW: 74.55 g mol^{-1}, VWR Chemicals). Absolute ethanol (99.9%, C_2H_6O, MW: 46.07 g/mol, VWR Chemicals) was used to clean silicon wafers prior to the AFM characterization.

Mouse anti-human cardiac troponin-I monoclonal antibody (4.7 mg mL^{-1}, liquid in PBS, pH 7.4, with 0.09% sodium azide, isotype IgG_1, Sigma Aldrich, Germany) and cardiac troponin-I (cTnI, MW: 23.9 × 10^3 g mol^{-1}, Sigma Aldrich, Germany) were selected as the model bioreceptor and target analyte, respectively for the immunoreaction. Analyte detection was performed using hydrogen peroxide (35%, H_2O_2, MW: 34.01 g mol^{-1}, Carl Roth) via amperometric measurements based on the electro-catalytic activity of as-mentioned nanomaterials. After antibody immobilization, the unreacted carboxyl groups of GQDs were blocked by bovine serum albumin (BSA, ≥98%, pH 7.0, Sigma Aldrich). The cross-reactivity studies were investigated by comparing the response to our target binding with respect to other interfering compounds such as neuron-specific enolase from human brain (NSE, ≥95%, SDS–PAGE, Sigma Aldrich), D–(+)–glucose (≥99.5%, $C_6H_{12}O_6$, MW: 180.16 g mol^{-1}, Sigma

Aldrich), BSA, transferrin from human blood plasma (≥95%, SDS–PAGE, Sigma Aldrich) and dopamine ($C_8H_{11}NO_2{\cdot}HCl$, MW: 189.64 g mol^{-1}, Sigma Aldrich).

Synthesis of gold nanoparticles (AuNPs)

AuNPs used for the fabrication of as-designed electrochemical immunosensor have been domestically synthesized by following the method, reported by Piella *et al.* [1]. A 75 mL of freshly prepared reducing solution of 2.2 mM sodium citrate containing 0.05 mL of 2.5 mM tannic acid and 0.5 mL of 150 mM potassium carbonate (K_2CO_3) was heated with a heating mantle in a three-necked round-bottom flask under vigorous stirring. When the temperature reached 70°C, 0.5 mL of 25 mM tetrachloroauric acid ($HAuCl_4$) was injected. The color of the solution changed rapidly to black-gray within 10 sec and then to orange-red in the following 1−2 min. The solution was kept at 70 °C for 5 min more to ensure complete reaction of the gold precursor. The resultant particles (∼3.5 nm, 7×10^{13} NPs mL^{-1}) were narrowly dispersed and negatively charged. The addition of 1 mM of K_2CO_3 in the reducing solution resulted in a pH ∼10, which decreased in the reaction mixture to pH ∼8 due to the introduction of $HAuCl_4$. This slightly basic value seems to have a meritorious effect resulting in narrower size distributions of the AuNPs.

While dealing with the reaction mixture, various critical parameters such as pH, temperature and tannic acid concentration were taken into a consideration. Herein, $HAuCl_4$, K_2CO_3, $C_{76}H_{52}O_{46}$ and $C_6H_5Na_3O_7.2H_2O$ act as reaction precursor, pH regulator, synthetic accelerator, and reducing as well as capping reagent, respectively. After the synthesis, different batches of AuNPs were stored at 4 °C by covering them with aluminium foil.

SPGEs and their cleaning procedure

SPGEs (8DS220AT, Metrohm, Germany) were used as an electrode system, which contained gold surface as a working area as well as auxiliary (counter) area, while silver as a reference material. The substrate material of SPGEs was composed of ceramic with dimensions 3.4 × 1.0 × 0.05 cm. The diameter and the geometrical area of the working electrode was 4 mm and 0.11 cm^2, respectively.

SPGEs were cleaned using the nitrogen plasma (Model: Henniker plasma HPT-200) for around 1 min, power: 50 W and pressure: 5×10^{-2} mbar [2]. They were then preserved in cleaned and sterilized glass petri-plates, tightly closed with para-film, prior to the immunoassays. Prior to performing the as-designated experiments, SPGEs were then baked in

a vacuum-oven at 120 °C, to get rid of all the possible contaminations as well as to eliminate the residual oxidized moieties. Subsequently they were rinsed with double distilled water and dried using 1 bar nitrogen gun.

Instrumentation and electrochemical analysis

Electrochemical (voltammetric, impedimetric and amperometric) measurements and characterization were carried out using the PalmSens 4 (software version PS Trace 5.8.1), a compact electrochemical interface with a three-electrode system connected to a DropSens device. For all the measurements, 40 μL was used as the sample volume and all the measurements were performed at room temperature.

Cyclic and square wave voltammograms as well as amperometric measurements were recorded in triplicate to confirm the reproducibility of the sensor signal during fabrication steps. **Table S1** depicts the parameters used for voltammetric, impedimetric and amperometric measurements, respectively. For the analyte determination, all these electrochemical methods (CV, SWV, EIS and amperometry) were separately applied on different SPGEs.

Supporting Tables and Figures

Table S1. Parameters for voltammetric, impedimetric and amperometric techniques.

CV method	SWV method	EIS method	Chronoamperometry
**Current range**			
1 μA – 10 mA	1 μA – 10 mA	1 nA – 10 mA	100 pA – 100 mA
**Pre-treatment settings**			
E condition = 0.0 V	E condition = 0.0 V	E condition 1 = 0.0 V	E condition = 0.0 V
t condition = 0 s	t condition = 0 s	t condition 1 = 0 s	t condition = 0 s
E deposition = 0.0 V	E deposition = 0.0 V	E condition 2 = 0.0 V	E deposition = 0.0 V
t deposition = 0 s	t deposition = 0 s	t condition 2 = 0 s	t deposition = 0 s
**Measurement settings**			
t equilibration = 2 s	t equilibration = 1 s	t equilibration = 0 s	t equilibration = 0 s
E begin = 0.8 V	E begin = - 0.3 V	Scan type = Fixed	E dc = - 0.2 V
E vertex1 = 0.8 V	E end = 0.8 V	E dc = 0.0 V	t interval = 0.1 s
E vertex2 = - 0.2 V	E step = 0.003 V	E ac = 0.01 V	t run = 200.0 s
E step = 0.004 V	Amplitude = 0.05 V	Frequency type = scan	
Scan rate = 0.1 V s^{-1}	Frequency = 10 Hz	N frequencies: 41 = 10/dec.	
Number of scans = 3		Max. frequency = 50000.0 Hz	
		Min. frequency = 5.0 Hz	

Table S2. Comparison of critical features offered by the as-designed AuNPs@GQDs/SPGE based electrochemical immunosensor for cTnI detection with respect to various other nanomaterial based electrochemical immunosensors reported so far for the cTnI detection.

Nanomaterials/ substrate	Electrochemical Detection technique(s)	Media	Investigation range	Limit of detection	Reference
AuNPs-modified ITO electrode	CV	Human serum	1–100 ng mL^{-1}; 100–500 ng mL^{-1}	1 ng mL^{-1}	[3]
SPE/ PdNPs	SWV	Human plasma	0.1–10 ng mL^{-1}	0.1 ng mL^{-1}	[4
SPE/ PtNPs	CV	Human plasma	0.1–55 ng mL^{-1}	0.07 ng mL^{-1}	[4]
Ch-$Ni_3V_2O_8$	CV, EIS	Human serum	0.005–100 ng mL^{-1}	5 pg mL^{-1}	[5]
GQDs/PAMAM	CV, DPV	Human serum	10^{-6}–10 ng mL^{-1}	20 fg mL^{-1}	[6]
**AuNPs@GQD/ SPGE**	CV, SWV, EIS, Amperometry	PBS	1–1000 pg mL^{-1}	0.1 pg mL^{-1}	**Current Work**
		Human Serum	10–1000 pg mL^{-1}	0.5 pg mL^{-1}	

Abbreviations: AuNPs: Gold Nanoparticles; Ch-$Ni_3V_2O_8$: Nickel vanadate hollow sphere modified chitosan; CV: Cyclic voltammetry; DPV: Differential pulse voltammetry; EIS: Electron impedance spectroscopy; GQDs: Graphene quantum dots; ITO: Indium tin oxide; PAMAM: Polyamidoamine; PBS: Phosphate buffer saline; PdNPs: Palladium nanoparticles; PtNPs: Platinum nanoparticles; SPE: Screen printed electrode; SPGE: Screen printed gold electrode.

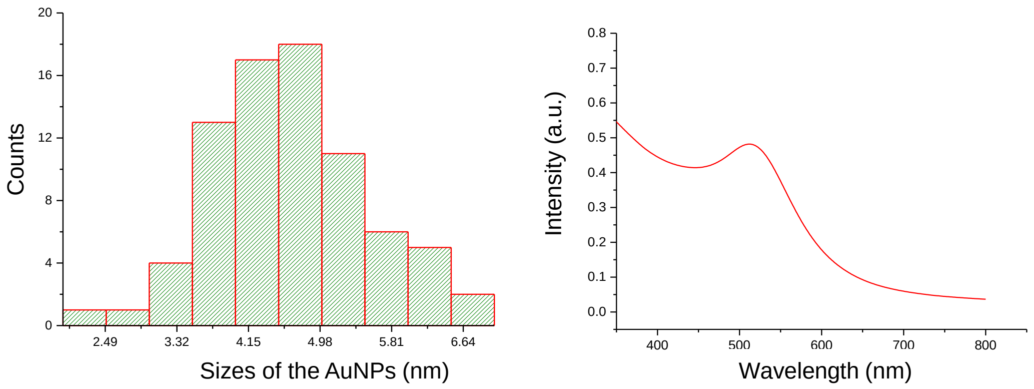

Fig. S1. (A) Size distribution histogram of AuNPs. **(B)** UV-Vis absorption spectrum of AuNPs.

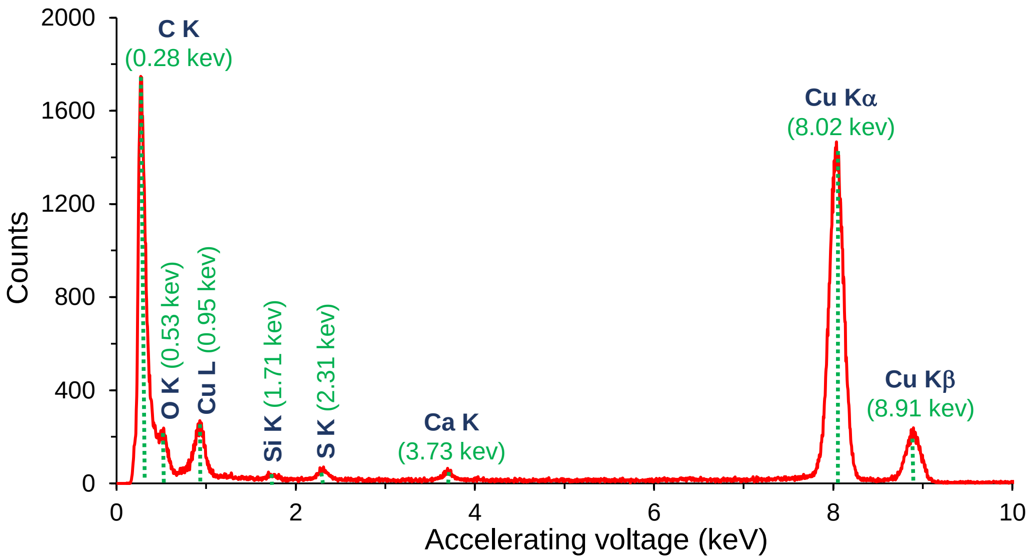

Fig. S2. EDX spectrum of GQDs. The dotted green lines corresponds to the accelerating voltage of their respective chemical elements.

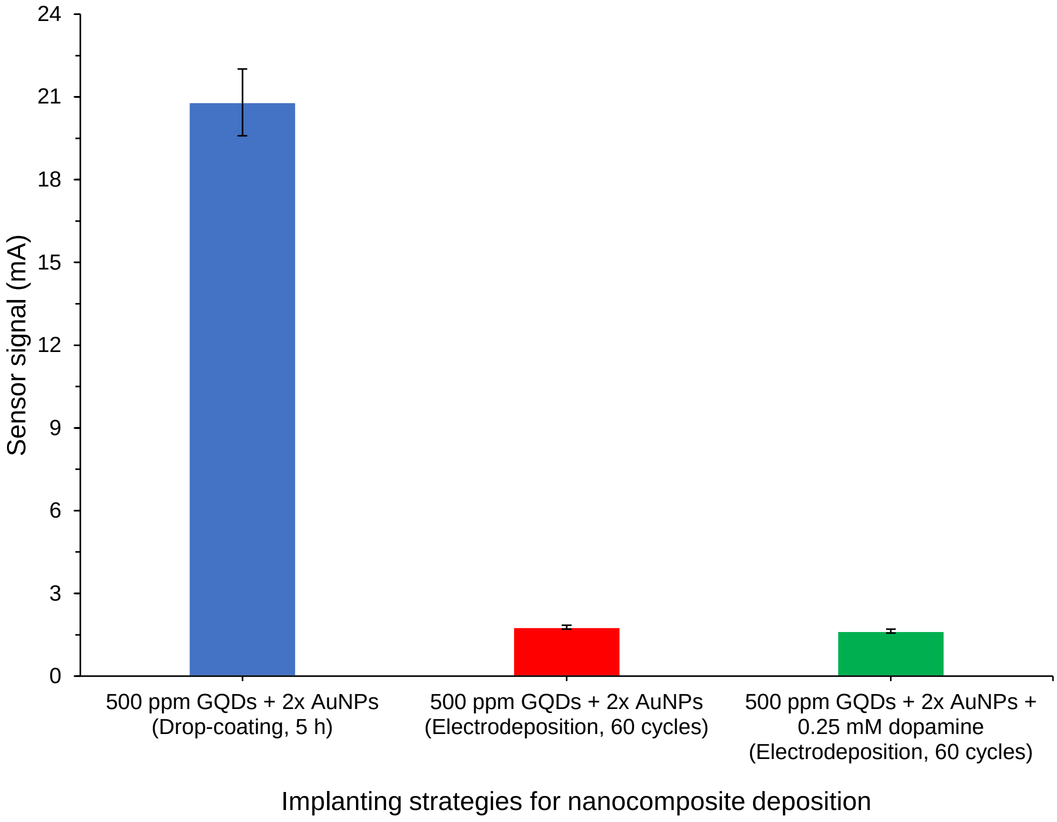

Fig. S3. Comparison of the signal response for different implanting approaches. 2× dilution of AuNP solution includes 2.13×10^{13} particles mL^{-1}. (n = 3)

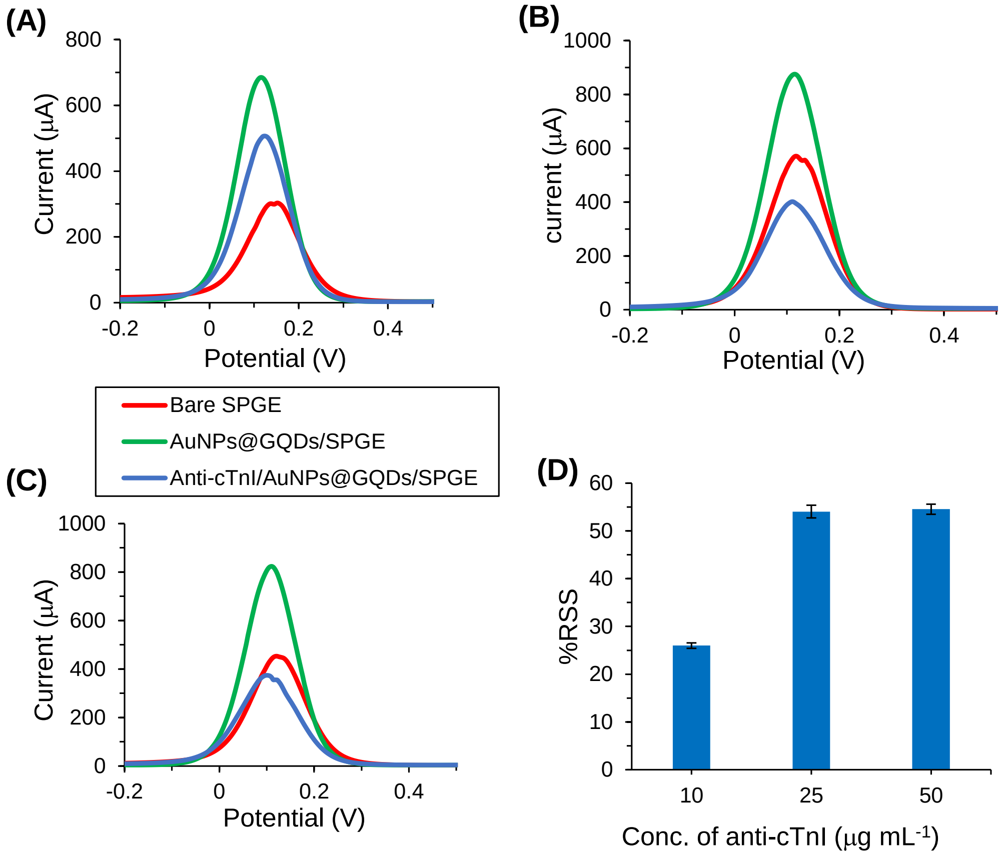

Fig. S4. Square wave voltammograms recorded using redox marker containing 10 mM $K_3[Fe(CN)_6]$ and 0.1 M KCl for the bare SPGE (red curve), AuNPs@GQDs/SPGE (green curve) and anti-cTnI/AuNPs@GQDs/SPGE (blue curve). Investigation of optimal anti-cTnI concentration **(A)** 10 µg mL^{-1} **(B)** 25 µg mL^{-1} **(C)** 50 µg mL^{-1}. **(D)** Comparison of the signal-concentration relationship between anti-cTnI/AuNPs@GQDs/ SPGE immobilized with 10 µg mL^{-1}, 25 µg mL^{-1} and 50 µg mL^{-1}. (n = 3).

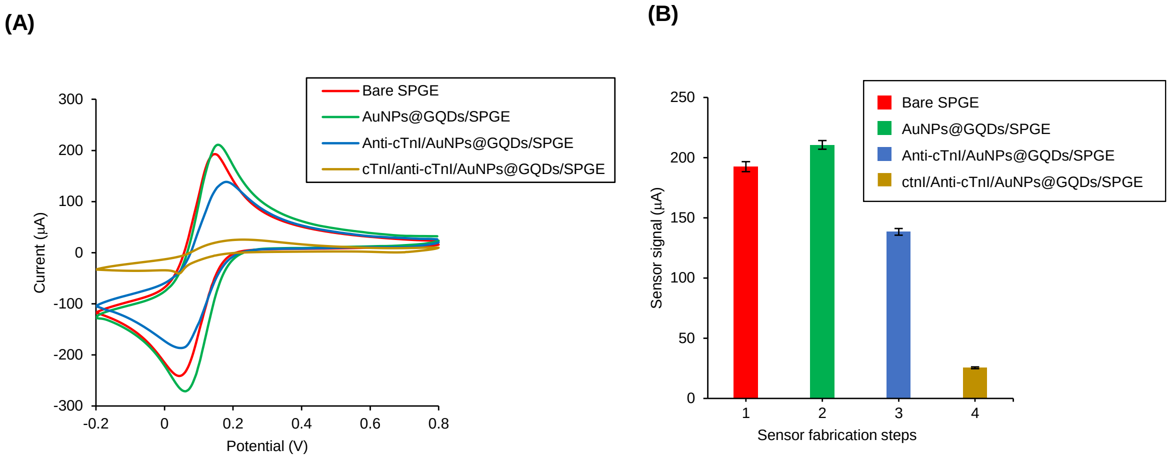

Fig. S5. (A) Cyclic voltammograms recorded using redox marker containing 10 mM $K_3[Fe(CN)_6]$ and 0.1 M KCl for the bare SPGE (red curve), AuNPs@GQDs/SPGE (green curve) anti-cTnI/AuNPs@ GQDs/SPGE (blue curve) and cTnI/anti-cTnI/AuNPs@GQDs/ SPGE (brown curve). **(B)** Comparison of the signal responses generated by each fabrication step. Step 4 involves 100 pg mL^{-1} cTnI binding on the nanocomposite sensor. (n = 3).

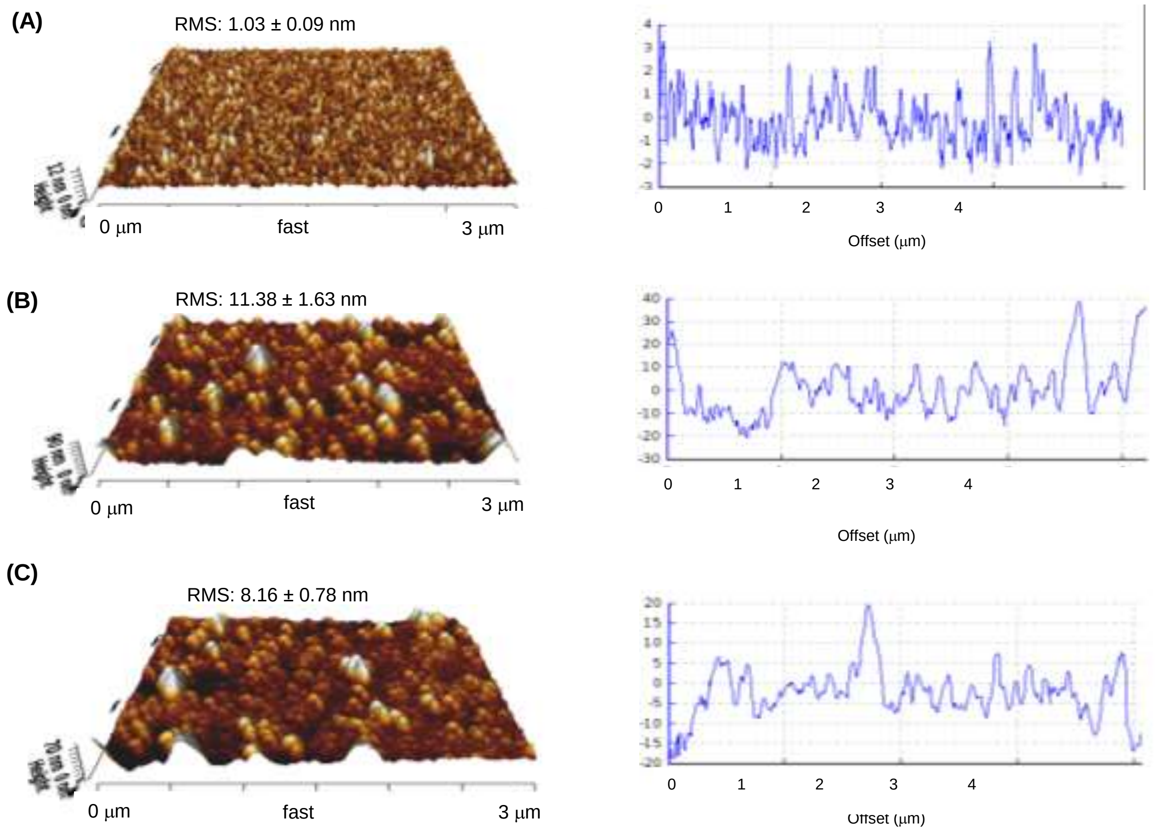

Fig. S6. AFM images for 3D surface topology images and cross-sectional height profile of **(A)** bare Si-wafer, **(B)** AuNPs@GQDs/Si-wafer and **(C)** anti-cTnI/AuNPs@ GQDs/Si-wafer at 3 µm × 3 µm imaging area. The RMS results are average of 10 cross-sectional data

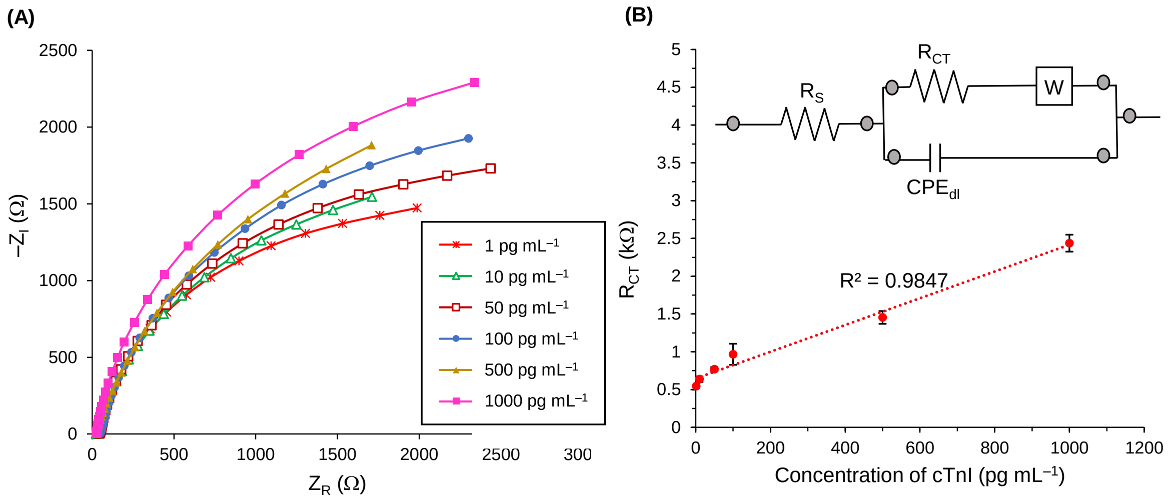

Fig. S7. (A) Nyquist plots recorded using redox marker containing 10 mM $K_3[Fe(CN)_6]$ and 0.1 M KCl for the target binding with anti-cTnI/AuNPs@GQDs/SPGE, by preparing six different concentrations of cTnI (1 – 1000 pg mL^{-1}) in PBS. **(B)** Linear regression showing charge transfer resistance (R_{CT}) w.r.t. different cTnI concentrations (n=3). The inset shows the circuit model employed for the curve fittings.

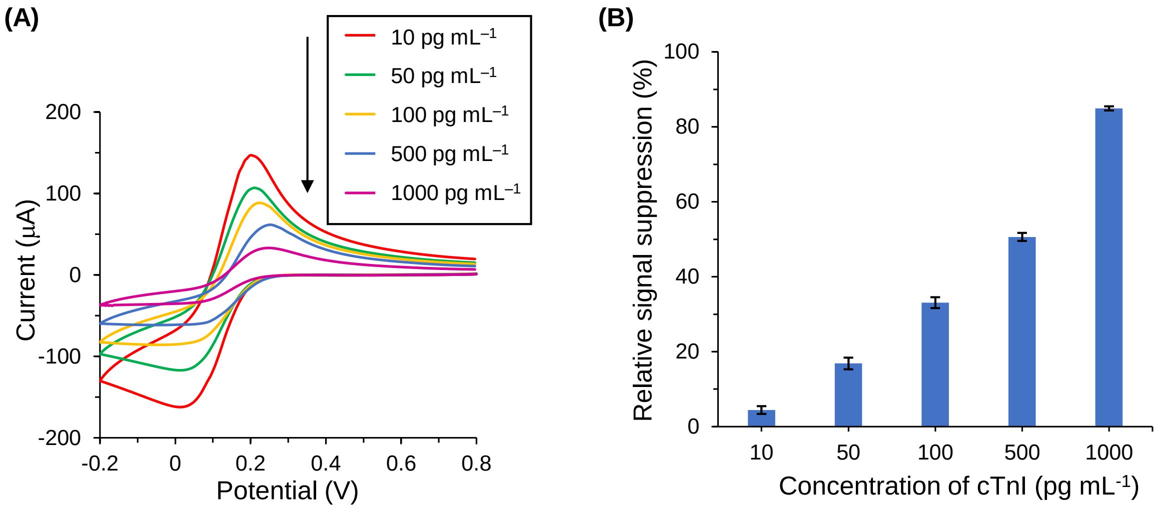

Fig. S8. (A) Cyclic voltammogram recorded using redox marker containing 10 mM $K_3[Fe(CN)_6]$ and 0.1 M KCl for the target binding with anti-cTnI/AuNPs@GQDs/SPGE, by preparing five different concentrations of cTnI (10 – 1000 pg mL^{-1}) in human serum. **(B)** Bar chart of concentration dependent cTnI bio-assay using CV-based detection method (n=3).

References

[1] Piella, J., Bastús, N.G., Puntes, V., 2016. Size-Controlled Synthesis of Sub-10-nanometer Citrate-Stabilized Gold Nanoparticles and Related Optical Properties. Chem. Mater. 28, 1066–1075. https://doi.org/10.1021/acs.chemmater.5b04406

[2] Altintas, Z., Uludag, Y., Gurbuz, Y., Tothill, I.E., 2011. Surface plasmon resonance based immunosensor for the detection of the cancer biomarker carcinoembryonic antigen. Talanta 86, 377–383. https://doi.org/10.1016/j.talanta.2011.09.031

[3] Ahammad, A.J., Choi, Y.H., Koh, K., Kim, J.H., Lee, J.J., Lee, M., 2011. Electrochemical detection of cardiac biomarker troponin I at gold nanoparticle-modified ITO electrode by using open circuit potential. Int. J. Electrochem. Sci. 6, 1906–1916.

[4] Xu, Y., Yang, S., Shi, W., 2017. Fabrication of an immunosensor for cardiac troponin I determination. Int. J. Electrochem. Sci. 12, 7931–7940. https://doi.org/10.20964/2017.09.65

[5] Singh, N., Rai, P., Ali, M.A., Kumar, R., Sharma, A., Malhotra, B.D., John, R., 2019. A hollow-nanosphere-based microfluidic biosensor for biomonitoring of cardiac troponin i. J. Mater. Chem. B 7, 3826–3839. https://doi.org/10.1039/c9tb00126c

[6] Bhatnagar, D., Kaur, I., Kumar, A., 2017. Ultrasensitive cardiac troponin I antibody based nanohybrid sensor for rapid detection of human heart attack. Int. J. Biol. Macromol. 95, 505–510. https://doi.org/10.1016/j.ijbiomac.2016.11.037
